# Awareness, knowledge, and risks of zoonotic diseases among livestock farmers in Punjab

**DOI:** 10.14202/vetworld.2015.186-191

**Published:** 2016-02-18

**Authors:** Jaspal Singh Hundal, Simrinder Singh Sodhi, Aparna Gupta, Jaswinder Singh, Udeybir Singh Chahal

**Affiliations:** 1 Department of Veterinary and Animal Husbandry Extension Education, Guru Angad Dev Veterinary and Animal Sciences University, Ludhiana, Punjab, India; 2 Krishi Vigyan Kendra, Ropar, Punjab Agricultural University, Ludhiana, Punjab, India

**Keywords:** awareness, knowledge level, livestock farmers, risk factors, zoonotic diseases

## Abstract

**Aim:**

The present study was conducted to assess the awareness, knowledge, and risks of zoonotic diseases among livestock farmers in Punjab.

**Materials and Methods:**

250 livestock farmers were selected randomly and interviewed with a pretested questionnaire, which contained both open and close ended questions on different aspects of zoonotic diseases, i.e., awareness, knowledge, risks, etc. Knowledge scorecard was developed, and each correct answer was awarded one mark, and each incorrect answer was given zero mark. Respondents were categorized into low (mean − ½ standard deviation [SD]), moderate (mean ± ½ SD), and high knowledge (Mean + ½ SD) category based on the mean and SD. The information about independent variables *viz*., age, education, and herd size were collected with the help of structured schedule and scales. The data were analyzed by ANOVA, and results were prepared to assess awareness, knowledge, and risks of zoonotic diseases and its relation with independent variables.

**Results:**

Majority of the respondents had age up to 40 years (70%), had their qualification from primary to higher secondary level (77.6%), and had their herd size up to 10 animals (79.6%). About 51.2% and 54.0% respondents had the history of abortion and retained placenta, respectively, at their farms. The respondents not only disposed off the infected placenta (35.6%), aborted fetus (39.6%), or feces (56.4%) from a diarrheic animal but also gave intrauterine medication (23.2%) bare-handedly. About 3.6-69.6% respondents consumed uncooked or unpasteurized animal products. About 84.8%, 46.0%, 32.8%, 4.61%, and 92.4% of livestock farmers were aware of zoonotic nature of rabies, brucellosis, tuberculosis, anthrax, and bird flu, respectively. The 55.6%, 67.2%, 52.0%, 64.0%, and 51.2% respondents were aware of the transmission of zoonotic diseases to human being through contaminated milk, meat, air, feed, or through contact with infected animals, respectively. The transmission of rabies through dog bite (98.4%), need of post-exposure vaccination (96.8%), and annual vaccination of dogs (78%) were well-known facts but only 47.2% livestock owners were aware of the occurrence of abortion due to brucellosis and availability of prophylactic vaccine (67.6%) against it as a preventive measure. About 69.2% respondents belonged to low to medium knowledge level categories, whereas 30.8% respondents had high knowledge (p<0.05) regarding different aspects of zoonotic diseases. Age, education, and herd size had no significant effect on the knowledge level and awareness of farmers toward zoonotic diseases.

**Conclusion:**

Therefore, from the present study, it may be concluded that there is a need to create awareness and improve knowledge of livestock farmers toward zoonotic diseases for its effective containment in Punjab.

## Introduction

Zoonoses, diseases and infections that are naturally transmissible between vertebrate animals and humans [1], are among the most frequent and dreaded risks to which mankind are exposed. The emergence and re-emergence of zoonoses and its potentially disastrous impact on human health are a growing concern around the globe [2]. Brucellosis, rabies, human African trypanosomiasis, bovine tuberculosis, cysticercosis, echinococcosis, and anthrax are listed as seven endemic zoonoses of concern [3]. In developing countries, they constitute an important threat to human health [4] especially for societies that domesticate and breed animals for food and clothing.

The Indian subcontinent has been identified as one of the four global hot-spots at increased risk for emergence of new infectious diseases (Public Health Foundation of India). The latest high-resolution climate change scenarios and projections for India (based on a regional climate modeling system known as Providing Regional Climates for Impact Studies, forecasts the likely increase in annual mean surface temperature by the end of the century from 2.5°C to 5°C and with warming more pronounced in the northern parts of India and a more than 20% rise in summer monsoon rainfall is projected which indicates a pronounced impact of zoonoses in future [5]. Hence, Veterinary Public Health has become a much more active field of enquiry in India and is involved with human health than that it was before.

The zoonotic diseases may be transmitted to livestock farmers through contamination during production, processing, and handling of food products of animal origin. About 68% of workforce in India is in close contact with domestic animals [6] and their activities, such as working with animals and in their sheds, improper disposal of waste from animal sheds, skinning of infected animals, slaughtering of diseased animals, disposal of infective material from the diseased animals, and poor personal hygiene practices, have been reported to be important risk factors. Lack of awareness about the occurrence of zoonotic diseases and their impact on public health have acted as a major hurdle in commencing adequate and effective control measures [7]. In our perspective dairy farming management, culture and eating habits and perception of farmers about zoonotic diseases and their prevention needs to be assessed as an understanding about awareness and practices of farmers can be a useful tool in developing and improving existing control measures [8]. Thus, the present study aimed at investigating risks of zoonotic diseases among livestock farmers and to assess their awareness and knowledge level toward zoonotic diseases.

## Materials and Methods

### Ethical approval

No ethical approval was required as it is a survey based study; however, after obtaining consent from all the participants involved in the study, the data were collected.

### Study site

Guru Angad Dev Veterinary and Animal Sciences University, Ludhiana act as a knowledge hub to the farmers of Punjab. The livestock farmers regularly visit the university for trainings, cattle fairs, learning new technologies, solution of livestock problems, treatment of diseased animals, purchasing of university publications, mineral mixture, bypass fat, uromin lick, etc., from all over the state.The present study was conducted based on data collected from livestock farmers who visited the University from different districts of Punjab between January 1^st^, 2015 and August 31^st^, 2015.

### Sampling size

250 farmers were selected randomly who visited the university and interviewed with a questionnaire.

### Data collection

The respondents were interviewed with a questionnaire contained both open and close ended questions on different aspects of zoonotic diseases, i.e., awareness, knowledge, risks, etc. The questionnaire had 14 questions to assess potential sources of infection to the farmers and 20 questions to test their awareness and knowledge level. The questionnaire was pre-tested on a few selected farmers, and the easiness of completion of the questionnaire and ambiguity of questions were noted and subsequently revised before a large-scale interview of the farmers. The information about independent variables *viz*., age, education, and herd size were collected with the help of structured schedule and scales.

### Statistical analysis

Knowledge scorecard was developed, and each correct answer was awarded one mark, and each incorrect answer was given zero mark. Respondents were categorized into three groups [9] based on the mean (9.53±0.19) and standard deviation (3.12) as a measure of check.

**Table:** 

Total score on knowledge	Knowledge category
Less than (mean − ½ SD)	Low
Between (mean ± ½ SD)	Moderate
More than (mean + ½ SD)	High

SD=Standard deviation

The data were analyzed by ANOVA [10] using the software package SPSS version 16 [11], and results were prepared to assess awareness, knowledge, and risks of zoonotic diseases and its relation with independent variables.

## Results

**Figure-1:**
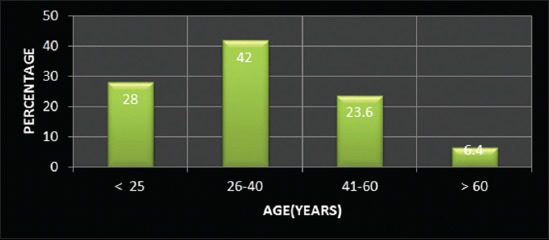
Distribution of respondents according to age.

**Figure-2:**
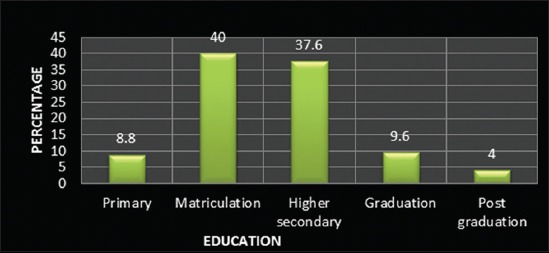
Distribution of respondents according to education.

**Figure-3:**
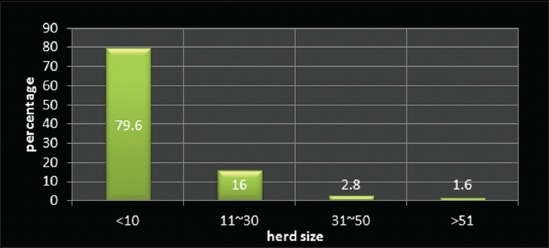
Distribution of respondents according to herd size.

The study revealed that 28% of farmers belonged to up to 25 year age category, and 42% belonged to 26-40 years age group, and rest 30% were of higher age groups (Figure-1) indicated that higher number of younger farmers were involved in the occupation of dairy farming. The education level of most of the farmers (77.6%) was up to matriculation or higher secondary, whereas merely 13.6% farmers were having a higher qualification (Figure-2). This is probably due to the fact that up to higher secondary level education is easier to be acquired at the local level. It was observed that 79.6% farmers in the selected population were small farmers with herd size up to 10 (Figure-3).

### Risk factors associated with conventional management and eating habits

The critical analysis of data revealed that around 51.2% and 54.0% respondents had the history of abortion and retained placenta, respectively, at their farm and the respondents not only disposed off the infected placenta (35.6%), aborted fetus (39.6%), or feces (56.4%) from diarrheic animal but also gave intrauterine medication (23.2%) bare handedly (Table-1). The majority of respondents assisted calving (80.4%) and did milking (93.6%) which could be a source of infection to them. As for as consumption of raw milk, egg, and meat is concerned, about 3.6 to 69.6% respondents not only consumed uncooked or unpasteurized animal products but also applied cream from raw milk on their skin cracks. Even sleeping in animal shed may be one of the risk factor associated with the occurrence of zoonotic diseases and about 30% respondents were following this practice. Newly purchased animal if suffered from diseases such as brucellosis or tuberculosis may act as a potential source of infection to farmers as well as to other animals, but merely 14% respondents got their animals tested for brucellosis and tuberculosis before making purchase.

**Table-1:** Exposure of livestock farmers to risk factors associated with various types of farm activities and eating habits.

Risk factors	Exposure	

	Frequency (n=250)	Percent
Eating habits		
Drinking raw milk	174	69.6
Eating raw meat	9	3.6
Eating raw eggs	65	26.0
Farm activities		
Milking	201	80.4
Sleeping in animal shed	75	30.0
Dealing with diarrheic animals	141	56.4
Assisting cow during calving	234	93.6
History of animal abortion at the farm	128	51.2
Disposed off aborted fetus with naked hands	99	39.6
Incidence of retained placenta	135	54.0
Disposed off placenta without bearing gloves	89	35.6
Intrauterine medication after abortion	58	23.2
Apply milk cream (raw milk) on cracks of lips	92	36.8
Testing of animal for brucellosis and tuberculosis before purchasing	35	14.0

### Awareness and knowledge of livestock farmers toward zoonotic diseases

On the basis of knowledge score, respondents were divided into low, medium, and high-level knowledge groups (Table-2). About 69.2% respondents belonged to low and medium knowledge level categories, whereas only 30.8% respondents had high knowledge regarding different aspects of zoonotic diseases. The differences were statistically significant (p<0.05) among all the groups.

**Table-2:** Knowledge level of livestock farmers toward zoonotic diseases.

Knowledge level	Frequency (n=250)	Percent
Low (upto 7.97 score)	71^a^	28.4
Moderate (7.98-11.09 score)	102^c^	40.8
High (≥11.10 score)	77^b^	30.8

Figures with different superscript in column differ significantly, p<0.05

As for as the awareness toward zoonotic diseases is concerned (Table-3), about 84.8%, 46.0%, 32.8%, 4.61%, and 92.4% of livestock farmers were aware of zoonotic nature of rabies, brucellosis, tuberculosis, anthrax, and bird flu, respectively, whereas as they had never heard about cysticercosis and echinococcosis. Even 92.8% of farmers listed swine fever among zoonotic diseases which may be due to the fact that media presented H_1_N_1_ as swine fever or swine flu in most of their reports.The zoonotic diseases may be transmitted to the human being through contaminated milk, meat, air, feed, or through contact with infected animals but this fact was known to 55.6%, 67.2%, 52.0%, 64.0%, and 51.2% respondents, respectively. Avian influenza virus may stick to the egg shell and may be enteredinto the food chain, but its transmission through raw egg was a lesser known fact (29.6%).

**Table-3:** Awareness of livestock farmers toward zoonotic diseases and their possible means of transmission.

Parameter	Frequency (n=250)	Percentage
Diseases transmit from animals to human being		
Rabies	212	84.8
Brucellosis	115	46.0
Bovine tuberculosis	82	32.8
Anthrax	12	4.61
Bird flu	231	92.4
Cysticercosis	0	0
Echinococcosis	0	0
Swine fever	232	92.8
Possible means of transmission of diseases from animals to human being		
Contaminating milk	139	55.6
Contaminating meat	168	67.2
Contaminating egg	74	29.6
Aerosol	130	52.0
Infected contaminating water or feed	160	64.0
Contact with infected animal	128	51.2
Awareness about rabies and brucellosis		
Rabies may result from		
Bite of rabid dog	246	98.4
Contact with rabid dog	17	6.8
From saliva of rabid dog	62	24.8
Rabid dog bite wound		
Wash with soap	173	69.2
Apply chili powder	77	30.8
Do we need vaccination after rabid dog bite	242	96.8
Vaccination of rabies in human		
Intra muscular	112	44.8
Intra peritoneal	138	55.2
Is annual vaccination of dog against rabies is necessary?	197	78.8
Brucellosis can cause abortion in dairy animals during which trimester of gestation period?	118	47.2
Is vaccination available against brucellosis?	169	67.6
Have you ever got disease transmitted to you from your animal	15	6.0

Rabies and brucellosis are the two most common diseases of zoonotic importance. The awareness level of farmers about rabies (Table-3) indicated that its transmission through dog bite was a well-known fact (98.4%) while it can also be transmitted through saliva (24.8%) and contact (6.8%) of infected dog were known to a lesser extent. About 69.2% respondents were aware ofthe use of soap to wash the wound immediately after dog bite but still 30.8% farmers opined to apply chili powder on it which is a mere misconception, and there is need to educate the people on this aspect. 96.8% respondents were aware ofneed of post-exposure vaccination in human, but 55.2% of them were still thinking that intra peritoneal was the only route of administration. Remarkably, 78% respondents were aware of annual vaccination of dogs for prevention of rabies. Brucellosis is another common disease of dairy animals which is zoonotic in nature and can cause economic loss as well as ahealth hazard to the farmers. However, only 47.2% livestock farmers were aware of the fact that animals may abort in the third trimester of their pregnancy due to brucellosis. Now-a-days, prophylactic vaccine is available for female dairy animals as a preventive measure, and about 67.6% of respondents were aware of it. When farmers asked about the disease/s that they acquired from their animals, about 6% respondents said yes, and it was a skin infection.

### Independent variables and knowledge level of farmers toward zoonotic diseases

The effect of age, education, and herd size on knowledge level and awareness of farmers toward zoonotic diseases was given in Table-4. The data revealed that age, education, and herd size didn’t affect the knowledge level and awareness of farmers toward zoonotic diseases as mean correct responses difference among different age, education, and herd size groups remained non-significant.

**Table-4:** Effect of age, education and herd size on knowledge level of livestock farmers toward zoonotic diseases.

Parameter	Mean correct responses	Significance
Effect of age (years) on knowledge level of livestock farmers		
≤25	9.56±0.40	NS
26-40	9.63±0.27	NS
41-60	9.17±0.43	NS
>60	10.12±0.87	NS
Effect of education on knowledge level of livestock farmers		
Primary	9.77±0.53	NS
Matriculation	8.93±0.30	NS
Higher secondary	9.85±0.31	NS
Graduation	10.96±0.77	NS
Post-graduation	8.60±1.27	NS
Effect of herd size on knowledge level of livestock farmers		
≤10	9.39±022	NS
11-30	10.45±0.51	NS
21-50	8.57±1.28	NS
>50	9.0±1.58	NS

NS=Non-significant, i.e., p>0.05

## Discussion

### Risk factors associated with conventional management and eating habits

Incidence of abortions, retained placenta, consumption of raw animal products, bare-handed handling of animal excreta and milking are the prime sources of infection [12]. The findings are in agreement with earlier results [13], who also reported similar practices of consumption of raw animal products. Researcher [14] observed that majority of the dairy farmers practiced hand milking. Ingestion of infected raw unpasteurized milk was cited as the most possible way of contracting milk-borne zoonoses [15].The unpasteurized or un-boiled milk have been reported to be associated with brucellosis and bovine tuberculosis [16-18]. Newly purchased animal if suffered from diseases such as brucellosis or tuberculosis may act as a potential source of infection to farmers as well as to other animals. Many of the respondents under study also followed these practices which may be due to the lack of awareness about the transmission of zoonotic diseases. The facts clearly indicated that the farmers were at high-risk end to get zoonotic diseases, and there is need to educate them about scientific management methods, safe disposal of infected material, and handling of livestock products for effective containment of zoonoses.

### Awareness and knowledge of livestock farmers toward zoonotic diseases

Healthy herd and health of livestock farmers both are equally important. However, the study indicated that knowledge level of livestock farmer was low to medium. This stressed on the need for providing better knowledge to them for effective control of zoonosis. As for as the awareness toward zoonotic diseases is concerned, awareness about rabies was high and findings are in agreement with another researcher [19], but awareness toward brucellosis, tuberculosis, and anthrax was low and even they had never heard the name of cysticercosis and echinococcosis diseases. Most of the farmers listed swine fever among zoonotic diseases which may be due to lack of awareness and printing of misinformation in a section of media. It not only creates a fear psychosis among pork consumers but also have huge economic impact on pig farmers as well as the nation. The zoonotic diseases may be transmitted to the human being through contaminated milk, meat, air, feed, or through contact with infected animals but this fact is not known to all of the farmers. Similar levels of knowledge were also reported by others [13] regarding the transmission of zoonotic diseases.

Like rabies, brucellosis is another disease of zoonotic importance which the livestock farmers may get from animals and clinically it may manifest as an acute or chronic form [20]. Awareness about rabies was good among livestock farmers, which may be due to the fact that dog bite is common in India due to a huge population of stray dogs and we always go for post-exposure vaccination. However, still the misconception like the application of chili powder on dog bite wound was there, which is need to be stressed. However, on other hand, farmers were not well aware of brucellosis as less than half of the respondents knew that *Brucella* can cause abortions in dairy animals. Now-a-days, prophylactic vaccine is available for female dairy animals as a preventive measure, but only two third farmers were aware of it. When farmers asked about the disease/s that they acquired from their animals, about 6% respondents said yes and it was the skin infection. It may be due to the reason that skin infection is visible easily, and other diseases cannot be diagnosed at farmer level. Some [21] also reported a low level of knowledge in respondents regarding zoonotic diseases. However, knowledge on rabies was found to be higher than other zoonotic diseases and this fact also conjoins with this study. Similar results were also reported by a researcher [22] where they concluded that 87% small scale holders had low to fair level of knowledge regarding zoonosis. This low and medium level of awareness could be due to remoteness, lack of health facilities, poor extension services, low training status on rearing and handling of animals, and low literacy rate which have been reported as major contributors to the low level of awareness among dairy farmers [23]. Now-a-days, improvement in the zoonotic diseases research should also be based newer basic science techniques and areas of genetic algorithms and ant colony optimization to combinatorial optimization problems [24-26].

### Independent variables and knowledge level of farmers toward zoonotic diseases

Age, education, and herd size didn’t affect the knowledge level and awareness of farmers toward zoonotic diseases significantly. It may be due to the reason that exposure to disease, training, and extension contacts might have played their role [23].

## Conclusion

Livestock farmers were well aware of rabies, but the knowledge toward other zoonotic diseases was low to medium. Even the farmers did not hear the name of cysticercosis and echinococcosis. Livestock holders were mostly not aware of the risk of contracting zoonotic pathogens from consuming contaminated raw milk, meat, and eggs. In addition, proper disposal of infected milk or dairy products, aborted materials, and use of hygienic procedures during milking and milk storage are extremely important steps in successful control of zoonotic pathogens [27]. These zoonotic diseases have a direct effect on human and animal health and production, but this may influence the economy of the country by being barriers to trade, increased cost of marketing the product to ensure it is safe for human consumption and the loss of market because of decreased consumer confidence. Inspite of its utmost importance, awareness to livestock farmers regarding their needs to be stressed on because due to lack of awareness most of them go undiagnosed and uncontrolled. Even though the government is practicing most disease control schemes including vaccination, organization of animal health camps but preponderance over the issue of improving awareness among the livestock owners could become a milepost in prevention and control of zoonotic diseases.

## Authors’ Contributions

JSH: Prepared, pretested and revised questionnaire for collection of data by personal interview and statistical analysis; SSS & AG: Provided valuable suggestions regarding the design of the study and analysis of the collected data; JS & USC: Provided guidance throughout the study period as well as helped in academic/legislative aspects. All authors read and approved the final manuscript.
